# Contributions of Mass Spectrometry-Based Proteomics to Understanding *Salmonella*-Host Interactions

**DOI:** 10.3390/pathogens9070581

**Published:** 2020-07-17

**Authors:** Buyu Zhang, Bohao Liu, Yinglin Zhou, Xinxiang Zhang, Qinghua Zou, Xiaoyun Liu

**Affiliations:** 1 Institute of Analytical Chemistry, College of Chemistry and Molecular Engineering, Peking University, Beijing 100871, China; 1601110334@pku.edu.cn (B.Z.); zhouyl@pku.edu.cn (Y.Z.); zxx@pku.edu.cn (X.Z.); 2 Department of Microbiology, School of Basic Medical Sciences, Peking University Health Science Center, Beijing 100191, China; 1710305339@pku.edu.cn (B.L.); zouqinghua@bjmu.edu.cn (Q.Z.)

**Keywords:** mass spectrometry, proteomics, *Salmonella*, host-pathogen interactions, AP-MS

## Abstract

As a model pathogen, *Salmonella* invades both phagocytic and non-phagocytic host cells and adopts an intracellular lifestyle in a membrane-bound compartment during infection. Therefore, a systemic overview of *Salmonella* adaptations to distinct host cells together with host remodeling will assist us in charting the landscape of host-pathogen interactions. Central to the *Salmonella*-host interplay are bacterial virulence factors (effectors) that are injected into host cells by type III secretion systems (T3SSs). Despite great progress, functional studies of bacterial effectors have experienced daunting challenges as well. In the last decade, mass spectrometry-based proteomics has evolved into a powerful technological platform that can quantitatively measure thousands of proteins in terms of their expression as well as post-translational modifications. Here, we will review the applications of high-throughput proteomic technologies in understanding the dynamic reprogramming of both *Salmonella* and host proteomes during the course of infection. Furthermore, we will summarize the progress in utilizing affinity purification-mass spectrometry to screen for host substrates of *Salmonella* T3SS effectors. Finally, we will critically discuss some limitations/challenges with current proteomic platforms in the context of host-pathogen interactions and highlight some emerging technologies that may offer the promise of tackling these problems.

## 1. Introduction

As a group of Gram-negative bacilli that parasitize in human and animal intestines, *Salmonella* can cause local intestinal disease (enteritis and diarrhea) in humans or, in some cases, systemic disease (enteric/typhoid fever). The group of typhoidal *Salmonella* contains serovars Typhi, Paratyphi A, Paratyphi B, and Paratyphi C, whereas other serovars are referred as non-typhoidal *Salmonella*, including *Salmonella* Typhimurium [1]. Here in this review, we focus on *Salmonella* Typhimurium which we will refer to as *Salmonella* unless otherwise indicated. As an intracellular pathogen, *Salmonella* can invade different types of human cells including epithelial cells and macrophages, which is essential for bacterial virulence. *Salmonella* pathogenesis is largely mediated by two type III secretion systems (T3SSs) encoded within *Salmonella* pathogenicity islands 1 and 2 (SPI-1 and SPI-2). These nanomachines are able to transport virulence factors (called effector proteins) directly into host cells to manipulate diverse cellular processes. The SPI-1 effectors mostly mediate the initial bacterial invasion into host cells, while those encoded within SPI-2 contribute to pathogen intracellular survival and multiplication. Upon internalization, *Salmonella* resides in a membrane-bound compartment known as *Salmonella*-containing vacuoles (SCVs).

As one of the model bacterial pathogens, *Salmonella* pathogenesis has been extensively studied by using various genetic, biochemical and cell biology methods. These approaches have built up most of our knowledge of *Salmonella* infection biology. Nevertheless, there are notable obstacles that hamper us from further understanding host-pathogen interactions on the molecular level. For instance, we often lack a systemic snapshot of the host-pathogen interplay in that the bulk of research has focused on characterizing individual pathogen genes/proteins. In addition, among the 40–60 *Salmonella* type III effectors identified so far, only a handful of those are well characterized in terms of their biochemical activities, host targets, and functional roles in pathogenesis. In reality, the function of many more effectors (particularly those encoded by SPI-2) are yet to be defined. Another unfortunate truth is that they are often more challenging to work with, or in other words, difficult to tackle by using traditional methods (otherwise they would have been found and studied already).

Recently, mass spectrometry (MS)-based proteomics has witnessed major technical breakthroughs. Owing to highly efficient nanoflow liquid chromatography (nano LC) and modern mass spectrometers with high sensitivity, throughput and resolution, current proteomic platforms have achieved enormous progress in analytical depth as well as the proteome coverage of complex samples. In the last decade, the number of detected proteins by LC-MS techniques has increased from 1000 to almost 10,000 in a single cell type [2].

We believe that it has come to the moment when MS-based proteomics can make substantial impact on the research of *Salmonella*-host interactions. Herein, we intend to summarize the utilization of proteomic technologies on elucidating the functional interplay between *Salmonella* and host cells. With respect to proteomic applications in the broader area of host-pathogen interactions (e.g., various bacteria and viruses), there are some excellent reviews published lately [3,4]. Furthermore, we would also like to discuss some pitfalls and/or limitations of current technologies in the context of the challenges they encounter. Last, we will envision some future directions of MS-based proteomics in shaping our understanding of *Salmonella*-host interactions.

## 2. Large-Scale Analyses of *Salmonella* and Host Proteomes during Infection

Bacteria in general can sense environmental cues and quickly adapt to their surroundings. Such adaptations are ultimately reflected in alterations on the molecular level and, in particular, the protein level. Therefore, the *Salmonella* proteome exhibits high plasticity upon interactions with its mammalian host. Conversely, host cells will have to reprogram their gene expression to fight against bacterial invasion and their intracellular proliferation [5]. Therefore, dynamic proteomes of both *Salmonella* and host cells upon infection would allow us to chart a panoramic view of the host-pathogen interplay and generate hypotheses that warrant further investigations.

### 2.1. Research on Salmonella Proteome during Infection

Given *Salmonella* as a facultative intracellular pathogen, it would be most informative to examine the proteome of bacteria isolated from infected host cells. For a long time, however, it had been technically challenging to physically isolate intracellular pathogens [6]. Therefore, a substantial body of work has focused on proteomic analyses of bacteria grown in bacteriological media or in conditions that mimic the host environment [7]. These studies have been extensively reviewed elsewhere [8]. Herein we will focus on proteomic analyses of *Salmonella* recovered from cultured cell lines or in vivo infection models.

The seminal work on the *Salmonella* proteome during infection was reported by the Bumann and Mann groups in 2006 [9]. Prior to this, such work had not been carried out for any pathogen as it is technically challenging to measure minimal amounts of bacterial proteins together with overwhelming excess of host contaminants. They managed to isolate bacterial pathogens from infected mouse tissues by using flow cytometry with an engineered *Salmonella* strain expressing green fluorescent protein (GFP). In total, they identified 370 and 835 *Salmonella* proteins from mouse spleen and caecum, respectively, most of which were metabolic enzymes nonessential for *Salmonella* virulence. In addition to in vivo studies, in the same year Shi et al. quantitatively analyzed the intracellular *Salmonella* proteome by using cell culture models, in which 315 bacterial proteins were identified from infected RAW 264.7 macrophages with 39 up-regulated proteins after infection [10]. Follow-up genetic studies established that STM3117 contributes to *Salmonella* proliferation inside macrophages. 

Given that *Salmonella* encodes ~4500 proteins, the proteome coverage of the above two studies still awaits further improvement to increase the analytical depth. In 2013, the Bumann group extended the previous work and detected 1182 bacterial proteins from an infected mouse spleen by using improved sorting and proteomic technologies [11]. They further focused on 477 metabolic enzymes, of which they performed absolute quantification and determined copy numbers. Eventually, they developed a computational model of *Salmonella* metabolism in infected host tissues and demonstrated that the pathogen exploits diverse host nutrients available only in scarce amounts. Though not reported in this study, it would be interesting as well to analyze bacterial proteins in the in vivo dataset other than metabolic enzymes (e.g., virulence factors, transcriptional regulators and etc.). In particular, quantitative assessment of the in vivo proteome together with control groups can be quite illuminating from the perspective of bacterial pathogenesis. 

**Figure 1:**
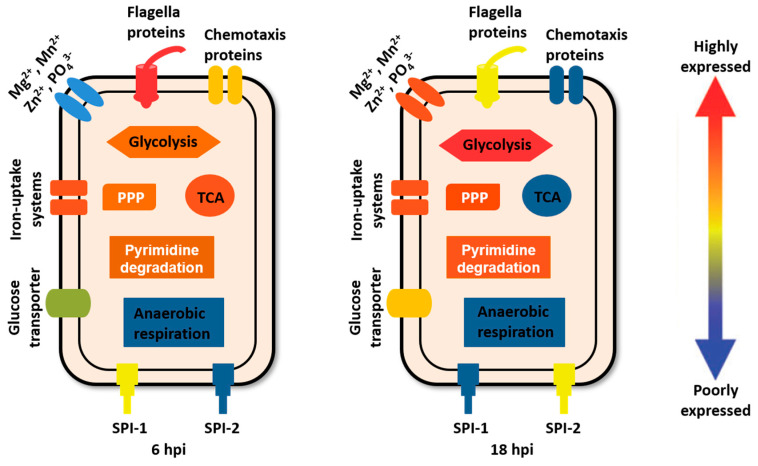
Figure adapted from Reference 14. Major proteomic signatures of intracellular *Salmonella* during infection of HeLa cells at 6 and 18 h post-infection (hpi). Relative protein expression levels of individual bacterial pathways are color-coded in the diagram.

Recently, we took advantage of a proteomic strategy developed previously for intracellular *Campylobacter* by the Galán group [12] and reported the dynamic remodeling of the *Salmonella* proteome within infected epithelial cells [13,14]. With optimized conditions for cell lysis and differential centrifugation, we detected close to 2000 bacterial proteins with minimal host contaminants (comprising <15% of all identified proteins). Intracellular bacteria were isolated at distinct stages of *Salmonella* infection (i.e., 1, 6 and 18 h post infection (hpi)) and their proteomes were quantitatively profiled together with extracellular populations. We reasoned that time-course analyses of the pathogen proteome are likely to reveal *Salmonella*’s adaptations to its intracellular niche within the host. At 6 hpi (the onset of bacterial replication), the most striking alteration was the massive induction of bacterial metal uptake (e.g., iron, zinc, and manganese) and phosphate utilization systems, unveiling an overall shortage of metal and phosphate within the SCV (Figure 1). At 18 hpi, the datasets revealed prominent reprogramming of bacterial metabolism. Notably, the tricarboxylic acid (TCA) cycle and respiration (both aerobic and anaerobic pathways) were repressed whereas glycolysis, the pentose phosphate pathway (PPP), and mixed acid fermentation were either constitutively expressed at high levels or further induced (relative to 6 hpi). Other proteomic signatures of intracellular *Salmonella* include differential regulation of virulence factors (i.e., mostly SPI-1 and -2 T3SSs) as well as severe degeneration of bacterial flagellar and chemotaxis systems (Figure 1). Overall, these large-scale proteomic datasets not only reveal broad adaptations of an intracellular pathogen to its mammalian host, but also provide us a rich resource for further studies of those protein hits with functions not well understood.

Among those differentially regulated proteins of intracellular *Salmonella*, YdcR, a putative transcriptional regulator, caught our attention because it was highly induced upon bacterial internalization into host cells (barely expressed extracellularly). To illuminate its potential roles in bacterial pathogenesis, in collaboration with the Song group, we again exploited intracellular bacterial proteomics [15]. Given its annotation as a transcription factor, we reasoned that contrasting the proteome of a mutant lacking *ydcR* (Δ*ydcR*) to that of its parental strain would reveal those candidate proteins under the regulatory controls of YdcR. Such a strategy turned out to be quite successful in nailing down the target proteins. The expression of SrfN, a known virulence factor, was found to be strictly dependent on YdcR during infection, and further biochemical assays validated the direct regulation of *srfN* by YdcR. Importantly, competitive infection reveals that the loss of YdcR largely phenocopied that of SrfN and led to reduced bacterial fitness in a murine infection model, thereby suggesting a physiological role in pathogenesis.

After breaching the intestinal epithelium, *Salmonella* can be further internalized by phagocytic cells, such as macrophages [16] where it disseminates to internal organs. Expectedly, *Salmonella* encounters different host environments in macrophages; therefore, it would be intriguing to uncover those differences to which pathogens further adapt. Our group profiled the intracellular *Salmonella* proteome inside infected murine macrophages (RAW 264.7 cells) [17]. Upon comparison to our previous data (infection of epithelial cells), many host-specific proteomic signatures were revealed for intracellular *Salmonella* (in addition to shared patterns). Notably, differential regulation of bacterial T3SSs, chemotaxis and flagellar systems showed substantially faster kinetics in macrophages relative to epithelial cells. Most strikingly, we noted massive induction of a complete set of *Salmonella* proteins encoded on *his* operon in RAW 264.7 cells while these bacterial enzymes were barely detectable during infection of HeLa cells. Follow-up experiments further established that markedly lower histidine levels in RAW 264.7 cells together with a *hisG* mutation of the strain (SL1344) drove up the abnormally high expression of the His enzymes. In other words, the defective biosynthetic pathway renders the bacterial strain hypersensitive to histidine shortage in host macrophage cells.

A recent study by the Hensel group employed intracellular bacterial proteomics in murine macrophages to study the roles of *Salmonella*-induced filaments (SIFs) [18]. While residing in the *Salmonella*-containing vacuoles (SCVs), *Salmonella* establishes an intertwined tubular network of SIFs connected to SCVs. The authors examined the proteome of two bacterial mutants, Δ*ssaV* and Δ*sseF*, which either lack a tubular network or have a defective one. In comparison to the WT strain, both mutants had elevated expression of bacterial proteins in response to nutritional starvation. In addition, the SPI-2-deficient mutant (Δ*ssaV*) exhibited features indicative of higher stress levels due to host antimicrobial defenses such as reactive oxygen species (ROS). These proteomic findings further support the roles of SIFs in the *Salmonella* intracellular lifestyle. Importantly, remodeling of the host endosomal system by SPI-2 T3SS effectors allows the pathogen to acquire nutrients and defend against host antimicrobial molecules.

### 2.2. Research on Host Proteome during Infection-Expression Profiling

Host-pathogen interactions are a two-way street. Surveying the host proteome can help understand host defense mechanisms during bacterial infection. Due to the greater complexity of mammalian proteomes, proteomic profiling of host cells, in general, is lagging behind that of pathogens. In 2009, Shi et al. reported the first proteomic analyses of host cells upon *S*. Typhimurium infection [19]. They conducted a time-course study of infected RAW 264.7 macrophages (at 0, 2, 4 and 24 hpi) together with uninfected controls. Among 1006 identified proteins, 244 host proteins were significantly altered after *Salmonella* infection. Notably, iNOS and prostaglandin (PG)-endoperoxide synthase 2 (also known as COX-2) were up-regulated, and their induction was previously shown to contribute to host defenses. Recently, our group examined protein expression of host epithelial cells (i.e., HeLa) during *Salmonella* infection [20]. In total, we measured 1558 proteins in nuclear fractions and 3916 proteins in post-nuclear fractions. Despite the increased proteome coverage, largely due to extensive sample fractionation, host proteomic datasets, at least in our hands, are yet to be as informative as those of pathogens. Nevertheless, host glucose transporters and glycolytic enzymes were markedly induced, which seems to resonate with elevated bacterial glycolysis as previously reported [14]. Other proteins of higher levels upon bacterial infection include integrins (e.g., ITGA2) and poly [ADP-ribose] polymerases (e.g., PARP1), yet their functional roles, if there are any, in *Salmonella*-host interactions remain to be defined.

Rather than profiling the whole proteome, subsets of the host proteome (called subproteomes) can be analyzed as well in response to bacterial infection. In principle, such analyses often afford greater details at the price of comprehensiveness. It has been shown that intact Golgi membranes are required for efficient *Salmonella* replication [21]. To investigate their roles, Vogels et al. analyzed Golgi-enriched fractions of infected HeLa cells and found 105 altered host proteins [22]. To further study the functional relevance, interestingly, small interfering RNA (siRNA) experiments identified five host factors (PRDX6, ITGB4, ERBB2IP, STOM and TBC1D10B), whose knockdown led to increased bacterial replication. *Salmonella* infection of host cells often accompanies the formation of extensive membrane networks extending from the mature SCV for nutrient supply. To understand the source and function of these networks, Vorweck et al. immunoprecipitated SseF, a SPI-2 effector embedded in such membranes [23]. Using a mutant lacking this membrane structure as a control, they found 247 specifically enriched host proteins, suggesting that *Salmonella* redirects ER membrane trafficking to its intracellular niche. 

Due to their vital function for intracellular *Salmonella*, SCVs have also been an intense subject of study, though their physical isolation is still technically challenging. Santos et al. examined the proteome of SCVs purified from HeLa cells at 30 min and 3 h, representing the early and maturing SCVs, respectively [24]. With non-infected fractions as controls, in total 392 host proteins were specifically enriched at SCVs. Interestingly, distinct sets of proteins were associated with the early and late stages, suggesting dynamic communications between diverse host organelles and SCVs (to promote bacterial intracellular survival). During *Salmonella* infection of macrophages, bacterial pathogens induce secretion of specific cytokines from these phagocytes and interfere with the host secretory pathways. For example, IL-4 and IL-10 suppress the host immune responses against *Salmonella*, while cytokines such as IL-1β and IL-18 are able to activate inflammasome. Hui et al. analyzed the extracellular proteome of macrophages (human THP-1 cells) upon *Salmonella* infection [25]. Their proteomic findings revealed for the first time the formation of proinflammatory exosomes in the early phase of macrophage infection, which can be further utilized to transport specific protein cargoes from infected cells to stimulate naive cells. Interestingly, one of these cargoes delivered via this pathway is a human deubiquitinating enzyme, OTUB1. 

In a recent study, the Typas and Krijgsveld groups performed host spatiotemporal proteomics during macrophage infection by combining click chemistry and pulsed stable isotope labeling of amino acids in cell culture (SILAC) [26]. They monitored newly synthesized host proteins across different infection stages and cell compartments (i.e., extracellular, lysosomal, and nuclear factions). Intriguingly, many lysosomal proteases (e.g., cathepsins) exhibited hypersecretion and higher levels in the nucleus. They further demonstrated that abnormal nuclear trafficking of cathepsins was mediated by *Salmonella* SPI-2 T3SS. Importantly, cathepsin inhibition was able to suppress *Salmonella*-induced cell death and reduce gasdermin D expression, thus illustrating a functional role of cathepsin in non-canonical inflammasome regulation. 

Lately, the Tao group developed a chemical probe that permits the capture of host proteins that directly interact with the surface of intracellular *Salmonella* during infection [27]. Briefly, the probe features three functionalities: a labeling aminooxy group that conjugates to the glycans on bacterial surface, a photo-reactive diazirine group that crosslinks host-interacting proteins and a biotin group for affinity purification. Murine macrophages (RAW 264.7 cells) were infected by labeled *Salmonella* and harvested at 15 min, 1 h and 6 h post infection prior to affinity enrichment of crosslinked host proteins. In total, 442 proteins were identified over three time points. Consistent with previous findings, host proteins associated with early endocytosis were enriched at 15 min and 1 h, while late endosomal and lysosomal proteins (e.g., LAMP1) were overrepresented at 6 h. In addition to known SCV markers and/or interacting host components, this work also identified novel substrates that bind to bacterial pathogens during early stages of infection, including CD98, CD180, CD147 and CD11b. It is conceivable that the developed strategy can be broadly applied to understand the interplay between hosts and other bacteria, as well as viruses.

### 2.3. Research on Host Proteome during Infection-PTM Profiling

Post-translational modifications (PTMs) play a crucial role in the interplay between pathogens and host cells. In the last decade, significant advances have been made in proteomic technologies towards large-scale profiling of several common PTMs such as phosphorylation, glycosylation, acetylation, and ubiquitination. Among the proteomic studies of all PTMs, phosphoproteomics is arguably the most established area in which diverse and robust enrichment strategies have been developed for phosphoproteins or phosphopeptides, such as immobilized metal affinity chromatography (IMAC) and TiO_2_ [28].

In 2011, the Foster group conducted quantitative phosphoproteomics of host epithelial cells during the initial stages of *Salmonella* infection [29]. They measured 1973 phosphorylated proteins and 9508 phosphorylation events from the analyses of host cells infected by wild-type and Δ*sopB* strains together with mock infection. Overall, they found higher phosphorylation levels of proteins related to regulation of apoptosis, transmembrane transport, nuclear organization, and cell proliferation and lower phosphorylation levels of those related to cytoskeleton organization, protein complex assembly, and cell polarity. Further bioinformatic analyses revealed that phosphosites of differing levels during infection were targeted by Akt, protein kinase C, and Pim. Moreover, they found that the SPI-1 T3SS effector SopB could impact half of the host phosphorylation events upon bacterial infection. In a subsequent study, the same group investigated the global impact of *Salmonella* SPI-2 effectors on the host phosphorylation events [30]. They quantitatively analyzed the phosphoproteome of epithelial HeLa cells and RAW 264.7 macrophage cells infected with either wild-type or SPI-2 T3SS-deficient strains (Δ*ssaV*). Intriguingly, they found differential modulation of host phosphorylation events and cellular processes by SPI-2 effectors in a cell type-dependent manner. Furthermore, they identified HSP27 as a kinase substrate of the type III effector SteC. Biochemical and cellular assays demonstrated that SteC induces actin rearrangement by phosphorylating multiple sites of HSP27.

In addition to phosphorylation, protein ubiquitination is central to many fundamental cellular processes and is also an important player at the functional interface of host-pathogen interactions. Indeed, the host ubiquitin system is a frequent target of many bacterial virulence factors [31,32]. For example, *Salmonella* encodes several T3SS effector proteins (e.g., SopA, SlrP, and SspH2), which harbor E3 ubiquitin ligase activity and presumably directly target host ubiquitination processes. The Behrends and Dikic groups utilized ubiquitinome profiling to measure the dynamic alterations in the global ubiquitination events of host epithelial cells upon *Salmonella* infection [33]. Overall, they found that host proteins of differing ubiquitination levels are functionally related to the actin cytoskeleton, NF-ĸB and autophagy pathways, and the Ub and RHO GTPase systems. Notably, pathogen-induced ubiquitination contributes to CDC42 activity and linear ubiquitin chain formation, both of which are necessary for the activation of NF-ĸB signaling pathways. 

Small ubiquitin-related modifier (SUMO) is a ubiquitin-like protein and SUMOylation can regulate a variety of protein functions in many cellular pathways [34]. The Srikanth group reported a dynamic host SUMOylation profile in response to *Salmonella* infection by using immunoblotting assays [35]. Moreover, they provided evidence that the intracellular survival of *Salmonella* was profoundly impacted by host SUMO status, suggesting a crucial role of this PTM in bacterial pathogenesis. In a following study, the same group enriched and analyzed the SUMOylated proteins from *Salmonella*-infected host cells as well as uninfected controls [36]. Among the diverse SUMO substrates, they followed up on the small GTPase Rab7 which was modified at K175. Furthermore, a SUMOylation-deficient Rab7 mutant exhibited longer half-life and was beneficial to SCV dynamics. Therefore, they concluded that the down-regulation of Rab7 SUMOylation by *Salmonella* promotes long-lived yet functionally deficient Rab7 for its own benefit of intracellular survival and proliferation. 

Recently, the Hartland group employed glycoproteomics to screen for host glycosylation substrates of *Salmonella* type III effectors SseK1 and SseK3. These two effectors are arginine glycosyltransferases that can covalently attach *N*-acetyl glucosamine (GlcNAc) to protein substrates. By taking advantage of an antibody that can recognize and enrich GlcNAcylated peptides, they aimed to globally identify glycosylated host proteins during *Salmonella* infection. They found that SseK1 modified the signaling adaptor TRADD, while SseK3 modified the signaling receptors TNFR1 and TRAILR [37]. In collaboration with the Li group, we applied the same strategy to comprehensively map the GlcNAcylated substrates of SseK3 upon bacterial infection. Our study revealed the modification of several Rab GTPases such as Rab1, consistent with the effector’s co-localization with the Golgi apparatus. Furthermore, the GlcNAcylated arginine residue was mapped to the switch II region and the third α-helix, and its modification severely disrupted the function of Rab1. Together, these studies demonstrate the utility of PTM profiling as a powerful screen for host targets of bacterial effectors. 

## 3. Proteomic Tools Assist in the Study of Bacterial Virulence Factors

In addition to the global profiling of protein expression and PTMs, MS-based proteomics has also evolved into a powerful technique for studying protein-protein interactions. In the context of *Salmonella* infection, secreted toxins and effectors are the bacterial factors that directly engage in host-pathogen interactions. Therefore, identifying these virulence factors and understanding how they work is a fundamentally important theme in bacterial pathogenesis. In fact, proteomic tools can make substantial contributions to such endeavors.

Over the years, many *Salmonella* type III effectors have already been reported by various biochemical, genetic, and bioinformatic methods. Nevertheless, we often lack a comprehensive survey of these effectors and whether we are still missing some effectors remains to be answered. Proteomic profiling of bacterial culture supernatant (or secretome analysis) can be utilized as a high-throughput screen for novel effector candidates. In 2011, the Heffron group analyzed bacterial secretome from *Salmonella* culture under SPI-2-inducing conditions [38]. By using the ∆*ssaL* mutant as a control, they were able to identify 20 known SPI-2 effectors. Among 12 candidate proteins for novel effectors, they successfully validated six proteins as bona fide substrates translocated by SPI-2 T3SS. Almost at the same time, the groups of Finlay and Foster collaboratively applied secretome analysis and identified several novel substrate candidates of SPI-2 T3SS as well [39]. In addition, they used quantitative mass spectrometry to examine the host targets of these bacterial effectors, which we will further discuss later in this section.

In 2017, our group undertook quantitative secretome profiling to catalogue *Salmonella* effectors delivered by SPI-1 T3SS [40]. By using a SPI-1-deficient mutant (∆*invA*) as a control, we identified STM1239 (which we renamed SopF) as a novel substrate of T3SS. Further immunoblotting and β-lactamase reporter assays confirmed T3SS-dependent secretion and translocation of SopF. In collaboration with the Zhou group, we performed some initial characterization of this newly identified effector, ascertaining its contribution to bacterial virulence. Recently, an independent study from the Shao group confirmed the discovery of SopF as a novel type III effector [41]. Remarkably, they unveiled that SopF inhibits *Salmonella*-induced autophagy (xenophagy) by covalently modifying a component of host vacuolar ATPase via its ADP-ribose transferase activity. We also contributed in this work to mapping the ADP-ribosylation site (s) of this host factor by tandem mass spectrometry.

As for bacterial virulence factors, a significant bottleneck of their functional studies is the discovery of host-interacting partners. When one embarks on such tasks, affinity purification-mass spectrometry (AP-MS) is arguably one of the most practiced methods. Indeed, AP-MS has helped uncover many host substrates of *Salmonella* effectors, which are scattered throughout the literature and include SopB, SopE, GogB, SteE, SifA, and SopA [42,43,44,45,46,47,48]. 

Next, we would like to review some reports in which large-scale AP-MS experiments were conducted to reveal host interactome for a panel of bacterial effectors. In 2011, the Finlay and Foster groups collaboratively screened a total of 24 SPI-2 effectors using strategies combining overproduction in mammalian cells or *E. coli*, immunoprecipitation of protein complexes, and MS analyses. In addition to previously described interactions, they were able to identify 11 novel protein interactions, three of which were further validated by reciprocal co-immunoprecipitation [39]. In 2016, another group reported AP-MS analyses of a subset of *Salmonella* effectors (GogA, GtgA, GtgE, SpvC, SrfH, SseL, SspH1, and SssB) and collectively identified 54 high-confidence host interactors [49]. Among those interactions, they further confirmed biochemically the interaction of SrfH and host kinase ERK2.

Despite the large body of the work discussed above, the overall success rate of AP-MS is not particularly encouraging, at least in some cases, in identifying host targets of individual effectors. There are two major complications, in our opinion, that could account for an unsuccessful practice. First of all, stable protein interactions/complexes (that can withstand and survive the whole purification scheme) have been assumed for positive outcomes. We know in reality that many effector-substrate interactions can be relatively weak or transient, which would be most likely lost during AP-MS experiments. Secondly, due to the sensitivity of MS measurements, one would pick up many non-specific binding partners (in particular when overexpression occurs) that mask the real targets from being identified. 

**Figure 2:**
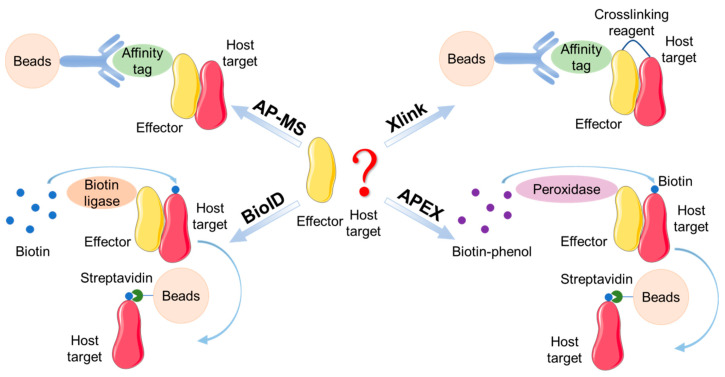
Affinity purification-mass spectrometry (AP-MS) strategies towards studying protein-protein (e.g., bacterial effector-host target) interactions and their emerging derivatives including BioID, APEX, and crosslinking MS.

Some emerging methods have shown promise in addressing the issues discussed above. Proximity labeling is one of these techniques. A prototype of this tool is BioID for proximity-dependent biotin identification [50]. Conceptually, BioID fuses a promiscuous biotin ligase with the bait protein (i.e., an effector) and upon expression in cells the fusion protein can biotinylate neighboring proteins in close proximity (Figure 2). Then, those biotin-labeled candidate interactors can be affinity purified under harsh conditions (to minimize non-specific interactions) for MS identifications. In principle, this approach also permits the isolation of weak and/or transient protein interactions. The Brumell group systematically compared BioID and traditional immunoprecipitation in analyzing host targets of *Salmonella* effectors that modulate host intracellular trafficking (SifA, PipB2, SseF, SseG and SopD2) [51]. Notably, BioID exclusively identified a subset of interacting proteins, among which SifA interacts with BLOC-2, a protein complex modulating dynein motor activity. Furthermore, they demonstrated the requirement of BLOC-2 for SifA-mediated positioning of SCVs. Their work therefore highlights the utility of BioID as a powerful tool to study effector–host protein interactions.

## 4. Concluding Remarks and Future Perspectives

As discussed above, proteomic analyses offer us a global and systemic view of the delicate balance of *Salmonella*-host interactions. Regarding proteome-wide expression profiling, the analyses of bacterial pathogens seem to provide us more insights into bacterial pathogenesis largely owing to relatively compact pathogen proteomes (and higher coverage). Though isolation of intracellular bacteria used to be technically challenging, we have perfected a differential centrifugation-based approach, permitting routine analyses of bacteria recovered from host cells. With such proteome datasets, we are able to chart a dynamic overview of pathogen biology during infection based on current knowledge of known protein functions. Nonetheless, we believe the better utility of such data is to formulate hypothesis, from which new studies can be initiated. We would like to raise attention in this regard because many altered proteins are not well characterized or cannot be classified into known pathways/networks (omitted from the main text). Furthermore, we would like to encourage the practice of analyzing intracellular proteome of bacterial mutants, particularly when a mutant fails to exhibit observable phenotypes in traditional assays. With WT bacteria as a reference, large-scale proteomic analysis of mutant strains can provide valuable clues by generating a molecular phenotype. Another future direction would be bacterial proteomes from in vivo infections, though we have seen a few successful examples as discussed early in Section 2.1. There are still some technical hurdles (e.g., limited amounts of samples, contamination from host and other resident bacteria) that we need to overcome.

On the host side, the proteome coverage in expression profiling has been expanded largely due to significantly improved proteomic platforms in the last decade. That being said, the covered proteome is still not as comprehensive as we would like it to be. In many cases, greater coverage comes with the price of extensive sample fractionations before LC-MS measurements, which substantially drives up the analysis time and lowers the throughput. Prior to the arrival of mass spectrometers with superb sequencing rates, we may have more success with subproteome analyses with reduced sample complexity and greater analytical depth. Subproteomes can be either organelles or other subcellular structures. Furthermore, PTMs can be considered as subproteomes as well. We have summarized some great applications of PTM profiling in the context of *Salmonella*-host interactions. In addition to generating networks of signaling pathways (e.g., phosphorylation) and new hypotheses, another direction we would like to encourage is the utilization of PTM profiling as a powerful screen for effector substrates. For example, *Salmonella* (and many other bacterial pathogens as well) encodes type III effectors harboring enzymatic activities that can directly engage in host phosphorylation and ubiquitination pathways. Quantitative PTM profiling of host cells infected by *Salmonella* WT and mutant strains can help us tease out candidate host targets. In the future, we would anticipate expansion of PTM profiling to other less common modifications when more enrichment tools become available. Though less prevalent than their mammalian counterparts, PTM profiling of bacterial pathogens can be explored as well. Indeed, large-scale analyses of protein acetylation in *Salmonella* have been reported [52,53].

A center stage of *Salmonella*-host interactions is the functional study of bacterial effectors. A common rate-limiting step in such endeavors is the relentless search for their host targets. Traditional AP-MS has fulfilled its duty to some extent in interrogating effector-target interactions, and smarter tools are definitely needed with the hope of better precision and accuracy. As discussed above, the efficiency of regular AP-MS would drop substantially when weak and/or dynamic protein-protein interactions are involved. Several emerging techniques can potentially overcome this difficulty including proximity-labeling methods such as BioID, as discussed above (Figure 2). A recently developed strategy involving engineered ascorbate peroxidase (APEX) can be exploited as well for proximity labeling and capturing candidate interactors [54,55]. APEX can convert biotin-phenol derivatives to short-lived radicals capable of reacting with electron-rich amino acids, thus biotinylating proteins within a small radius (<20 nm) (Figure 2). Lately, the Ting group further enhanced the labeling efficiency of BioID via directed evolution of the biotin ligase (now named TurboID) [56]. Thus far, proximity-labeling methods have contributed the discovery of several novel protein-protein interactions at the host-pathogen interface [57,58,59]. Last but not least, chemical crosslinking in principle can stabilize protein complexes by introducing covalent interactions. Indeed, we have successfully utilized formaldehyde crosslinking to pull down host targets of a *Legionella* type IV effector SetA harboring glycosyl-transferase activity [60]. Regardless of daunting challenges ahead, therefore, we should remain optimistic about constantly evolving new technologies. We will certainly envision in the future more prevalent practice of MS-based proteomics in unraveling molecular mechanisms governing the interplay of *Salmonella* and host cells as well as the broad field of host-pathogen interactions.
